# Sudden cardiac death in adults living with HIV: A systematic review

**DOI:** 10.1371/journal.pone.0334718

**Published:** 2025-10-30

**Authors:** James Pierzchalski, Nance Cunnigham, Katherine Kooij, Silvia Guillemi, Reginald Smith, Lawrence McCandless, Robert Hogg

**Affiliations:** 1 Faculty of Health Sciences, Simon Fraser University, Burnaby, British Columbia, Canada; 2 Walk With Me, Comox, British Columbia, Canada; 3 British Columbia Centre for Excellence in HIV/AIDS, Vancouver, British Columbia, Canada; 4 Faculty of Medicine, University of British Columbia, Vancouver, British Columbia, Canada; 5 Island Health Authority, Victoria, British Columbia, Canada; Keimyung University, KOREA, REPUBLIC OF

## Abstract

**Background:**

People living with HIV (PLWH) have rising life expectancy, and robust evidence shows they are also at increased risk of cardiovascular disease. However, sudden cardiac death (SCD) for PLWH on antiretroviral therapy (ART) has received little attention. Our systematic review examines the quantitative adult PLWH SCD risk literature with a sub-focus of PLWH on ART.

**Methods:**

We conducted systematic searches of PubMed, Embase, CENTRAL, CINAHL, Scopus, and Clinicaltrials.gov for peer-reviewed population studies using search terms “sudden cardiac death” AND (”HIV” OR “human immunodeficiency virus”) until 20 June 2025. Two reviewers analysed papers meeting eligibility criteria for their SCD classification methodology including for, but not limited to, comparability, generalizability, and misclassification biases including using the Newcastle-Ottawa Scale.

**Results:**

The eight eligible studies included ~98 436 PLWH and demonstrated that males PLWH experience elevated SCD risk compared to the general population. One study with 97% male participants found a hazard ratio (HR) of 1.14 (95% CI: 1.04–1.25) for PLWH compared to non-PLWH. In another, comparing viral load groups of ≥500 vs < 500 found a HR of 1.33 (95% CI: 1.04–1.71) for PLWH with CD4 ≥ 500 compared to HIV-negative HR of 1.03 (95% CI: 0.90–1.18). An autopsy study’s male sex arm found a mortality rate ratio for PLWH compared to a reference of 1.34 (95% CI: 0.62–2.87).

**Conclusions:**

The limited available research provides evidence that while SCD risk for male PLWH is elevated, maintaining HIV-RNA plasma viral load suppression and ≥200 CD4+ cells/mm^3^ counts (ideally higher) likely lowers the risk of SCD to a rate that is approaching comparability to the general population. The risk of SCD in women living with HIV is still unknown, due to small sample sizes, as the majority of the participants in the PLWH studies were male.

## Introduction

Population studies have demonstrated that approximately 50% of cardiovascular deaths could result from sudden cardiac death (SCD) [1,2]. SCD is a death subsequent to a sudden cardiac arrest (SCA) in a patient with known or previously undetected cardiac abnormalities where the mode and time of death are unexpected [3].

With antiretroviral therapy (ART) steadily improving, the life expectancy of people living with HIV (PLWH) has risen. However, longer life has led to their health burden partially shifting to cardiovascular diseases such as, but not limited to, myocardial infarction, heart failure, and stroke [4]. The burden of SCD in PLWH is likely also elevated but the estimates of mortality are not well defined or understood for this population [5]. Therefore, it is important to understand if PLWH are at an elevated risk of SCD compared to the general population and what factors may decrease the risk of SCD.

The challenge and difficulty of managing individual SCD risk is exemplified by the first clinical event for approximately 50% of SCD cases, is the presentation of cardiac arrest [3]. In a Canadian and USA site study where SCA occurred outside of a hospital setting and the patient received treatment by emergency medical services (n = 11 898) before being taken to the hospital, only 4.6% of patients survived and were subsequently discharged from the hospital [6]. SCD mainly occurs in the community setting; however, SCD is also a burden in the hospital setting [2,7]. A large (n = 84 625) study investigating SCA in hospitals estimated that ~77.7% of patients experiencing SCA did not survive [7].

The burden of sudden cardiac death (SCD) in PLWH is likely elevated compared to the general population due to HIV causing cardiovascular pathological changes. However, SCD in PLWH is not well understood nor has the risk been reliably quantified [5]. Despite the likely elevated risk of SCD for PLWH, we have limited knowledge about the risk of SCD for PLWH who are on ART. Our knowledge is also limited about HIV-specific causes of SCD.

The objective of this study was to complete a systematic review of the quantitative adult PLWH SCD risk literature with a sub-focus of PLWH on ART.

## Methods

### Information sources and searching

We performed systematic a searches of PubMed, CENTRAL, CINAHL, Embase, Scopus, and Clinicaltrials.gov using the terms ““sudden cardiac death” AND (”HIV” OR “human immunodeficiency virus”)”. The broad nature of the search terms was due to the limited literature and knowledge concerning the risk of SCD for PLWH; therefore, a wide search approach was employed.

### Eligibility criteria

To be eligible for this systematic review, we required articles to be peer-reviewed, cohort studies, randomized control trials, or single arm trials, deal with adult PLWH and SCD, provide a population risk or effect measure of SCD for PLWH in a well-defined population and be published before 20 June 2025. Additionally, the paper had to provide a clear methodology for its SCD classification. The review excluded papers that did not address HIV and SCD, were not peer-reviewed, or were case studies.

### Data collection and analysis

The review authors (JP & NC) independently assessed study eligibility, risk of bias, obtained effect measures, participant numbers, and assessed certainty of evidence. The Newcastle-Ottawa Scale was used as a tool to provide a supplemental systematic approach to the authors’ assessments [8]. All differences were resolved with discussion. Cochrane ‘Risk of bias’ tool to assess risk of bias in randomized studies, and ROBINS‐I tool for assessing risk of bias in non-randomized studies of interventions were not used, as the systematic review portion of the paper did not include randomized studies or measures of interventions [9,10]. While there is a relatively consistent definition of SCD, there have been conflicting estimates of adult SCD based on differing SCD ICD classifications [3]. This in turn means there is heterogeneity among study results. This was taken into account when considering possible biases with all studies having their SCD classification method reviewed. All papers that met eligibility criteria were analyzed for their SCD classification methodology, including, but not limited to, comparability, generalizability, and misclassification biases.

### Definition of SCD

SCD is defined as an unexpected death that has occurred as a result of sudden cardiac cause whereby a sudden loss of consciousness accrues within one hour of the onset of acute cardiovascular change of status [11]. Pre-existing heart disease is not an exclusion for SCD; however, the time and mode of death must be unexpected [11]. Significant discrepancies between SCD rates in the literature are due to the differences in SCD definitions used and the location of the study’s population [3]. Definitions range from only using coronary heart disease-related deaths to using almost all known causes of SCD [3]. This review utilizes Myerburg’s and Goldberg’s work on conflicting estimates of adult SCD based on differences in SCD ICD classification [3].

## Results

### Study selection

**Fig 1:**
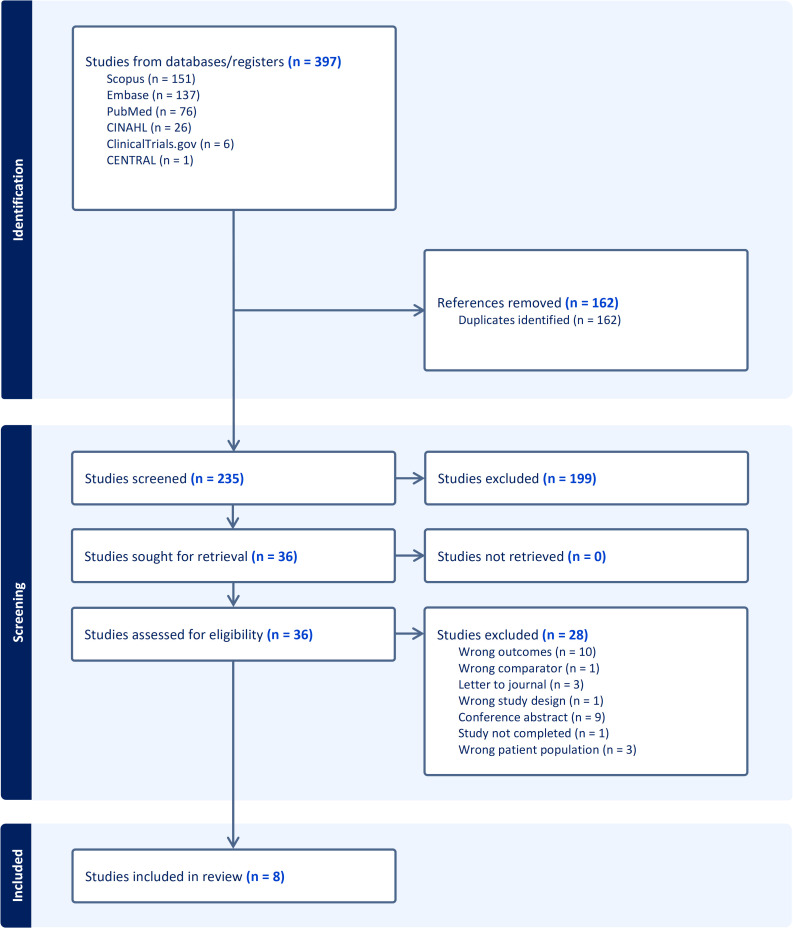
Identification of studies for inclusion for systematic review of PLWH population risk of sudden cardiac death.

Fig 1 provides a visual representation of the study selection using the methodology for information search. Of the 397 articles screened, eight met the study criteria.

### Systematic review of PLWH population risk of sudden cardiac death

The eight articles published before 20 June 2025 that specifically investigated SCD among PLWH are summarized in Table 1.

**Table 1:** Sudden cardiac death population studies summary for persons living with HIV.

Study	Period	Population	Outcome	Newcastle-Ottawa Scale/Biases
Tseng et al. 2012 [12]	April 2000 to August 2009	2 860 PLWH (93% male) in a San Francisco, USA clinic compared to a previous SCD study in San Francisco.	PLWH in a San Francisco HIV clinic had an SCD rate of 2.6 (95% CI: 1.8–3.8) per 1000 person-years. This is 4.5 times higher than expected from a general population study.	⋆⋆⋆ ⋆⋆⋆ ⋆⋆⋆ 9/9 Misclassification of some SCD – likely occult overdoses classified as SCD.
Moyers et al. 2014 [17]	April 2000 to August 2009	423 PLWH (81% male) subset of the Tseng et al. study of persons with at least one transthoracic echocardiographic evaluation	SCD with an EF of 40–50 (n = 4) HR of 1.9 (95% CI: 0.3–10.9), 30–39 (n = 3) HR of 9.5 (95% CI: 1.7–53.3), and <30 (n = 3) HR of 38.5 (95% CI: 7.6–195.0).	⋆⋆⋆ ⋆⋆⋆ ⋆⋆ 8/9 Possible misclassification of some SCD – likely occult overdoses classified as SCD.
Alvi et al. 2019 [18]	2011 Hospitalizations	2 149 patients (51% male) with hospitalization records from a single USA academic hospital with 344 PLWH (48% male).	Proportion of SCD over study period for HIV- patients was 6.4%, PLWH with CD4+ counts ≥200 cells/mm3 8.4%, and those with CD4+ counts <200 cells/mm3 were 37%. No CI reported for rate comparison except a 3.0 adjusted OR (95% CI: 1.78 to 4.24) for SCD rate of PLWH vs HIV- patients.	⋆⋆⋆ ⋆⋆⋆ ⋆⋆⋆ 9/9 Low generalizability to the population without known cardiac risks as patients already had a history of heart failure, as per the study design.
Lai et al. 2018 [19]	2000-2014	26 272 PLWH (aged 15 years and older, 93.9% male) from the Taiwan CDC HIV surveillance database from 2000 to 2014. The control group consisted of 2 million individuals randomly sampled.	SCD standardized incidence rate of 3.01 (95% CI: 2.39–3.73) compared to the general population, male PLWH 2.84 (95%CI: 2.23–3.56), and female PLWH 6.78 (95% CI: 2.92–13.36).	⋆⋆⋆ ⋆⋆⋆ ⋆⋆ 8/9 Likely substantial misclassification bias by including only three ICD codes, with two of the codes being non-cardiac specific and possibly including deaths due to overdoses. The authors state that the distributions of PLWH to control had no significant difference in age or sex; however, how this could occur randomly with a 93.9% male population is unclear.
Yen et al. 2019 [20]	2003-2014	24 306 PLWH (aged 15 years and older, 93.81% male) from the Taiwan CDC HIV surveillance database from 2003 to 2014. Matched controls 1:4 selected from the Taiwan National Health Insurance Program.	Hazard ratio PLWH vs HIV-negative 8.15 (95% CI: 5.58–11.9) Receiving ART vs those not receiving ART SCD was 0.53 (95% CI: 0.32–0.87). For 150 SCD events, 97 (64.7%) occurred in PLWH and 53 (33.3%) in controls.	⋆⋆⋆ ⋆⋆⋆ ⋆⋆⋆ 9/9 Likely substantial misclassification bias by including only three ICD codes, with two of the codes being non-cardiac specific and possibly including deaths due to overdoses.
Tseng et al. 2021 [21]	Feb. 2011 to Sept. 2016	15 979 PLWH (92% male) and 717 884 Adult Population (51% male) of San Francisco County, USA.	Male confirmed sudden death from arrhythmia PLWH had a 26.5 (16.6–40.1 95% CI) per 100 000 person-years and 19.8 (95% CI: 17.2–22.7) per 100 000 person-years for adult persons without known HIV infections for a 1.34 (95% CI: 0.62–2.87) incidence rate ratio.	⋆⋆⋆ ⋆⋆⋆ ⋆⋆⋆ 9/9 Did not adjust for ART status, CD4+ or VL. Limited female sex data.
Freiberg et al. 2021 [22]	Apr 2003 to Dec 2014,	43 407 PLWH vs 100 929 without HIV both 97.2% male. Match 1:2 by age (5-year interval), sex, race/ethnicity, & location.	PLWH vs HIV-negative persons SCD risk of 1.14 (95% CI: 1.04–1.25); though with adjusted SCD risk with time-varying HIV biomarkers PLWH CD4+ ≥ 500 the risk was 1.03 (95% CI: 0.90–1.18) and for VL < 500 0.97 (95% CI: 0.87–1.09).	⋆⋆⋆ ⋆⋆⋆ ⋆⋆⋆ 9/9 Not a true time varying analysis as it did not take into account history of CD4+ and VL as confounders.
deFilippi et al. 2025 [23]	March 26, 2015, to July 31, 2019	7 769 PLWH (69% male) in the REPRIEVE trial who were between 40–75 old, without known CVD and at low-to-moderate 10-year ASCVD risk treated with pitavastatin or a placebo	In the combined group there were 25 SCDs for a mortality rate of 0.61 per 1 000 person years. 14 of those SCDs were in the placebo control group compared to 11 in the pitavastatin group.	⋆⋆⋆ ⋆⋆⋆ ⋆⋆ 8/9 (adapted for cohort drawn from RCT) The study is underpowered due to being a subset analysis, so the authors did not perform a comparison between the pitavastatin and placebo groups.

Tseng et al. 2012, using a mixed-method and heterogeneous population, reported a 2.6 (95% CI: 1.8–3.8) per 1 000 person-years mortality rate at a San Francisco PLWH clinic cohort. This was 4.5 times the expected SCD rate, compared to a previous study investigating general population SCD in San Francisco [12]. The study used the International Classification of Diseases 10^th^ Revision (ICD-10) for its SCD case definition, which included ill-defined causes of death (R95-R99) [12]. If a death had an R99 code (other ill-defined and unspecified causes) and the person is was within ages 20–64, the death is likely an occult drug overdose, not an SCD [13,14]. In a general population study investigating out-of- hospital cardiac arrests in San Francisco from February 2011 to March 2014 (covering a period slightly after the conclusion of the Tseng et al. 2012 study) researchers found that of 525 persons autopsied, 16% were occult overdoses with a median age of 56 and interquartile range (IQR) of 49–62 compared to the non-overdose group, median age of 64 and IQR of 55–75 [15]. An additional reason for the difficulty in identifying SCD is that an ICD gold standard has not yet been developed [16].

Moyers et al. used a subset of Tseng et al. 2012 study using 423 PLWH with at least one transthoracic echocardiographic evaluation to evaluate PLWH ejection fractions (EF) on the risk of SCD. Of the 423 PLWH, 13 experienced SCD. PLWH hazard rates (HR) were compared against those with a greater than 50% EF (n = 3). There was a progression of increasing HR of SCD as EF decreased. For an EF of 40–50 (n = 4) HR of 1.9 (95% CI: 0.3–10.9), 30–39 (n = 3) HR of 9.5 (95% CI: 1.7–53.3), and <30 (n = 3) HR of 38.5 (95% CI: 7.6–195.0). In addition to the small sample issue, the study has the same misclassification bias potential as Tseng et al. 2012 [17].

Over a median 19-month follow-up period, Alvi et al. investigated the risk of SCD of PLWH who had a history of being hospitalized for heart failure (HF) (n = 344) with an HIV-negative control group (n = 1 805). Uniquely among these studies the women were balanced with the men; in the PLWH arm 52% were female (n = 178) as were 48% in the control group (n = 876) [18]. For PLWH with CD4+ counts ≥200 cells/mm^3^ (n = 189) with a history of HF, the proportion of SCD was similar to non-HIV persons at 8.4% to 6.4% (p = 0.31) [18]. PLWH with undetectable viral loads (VL) were found to have a comparable SCD proportion of 9%. However, within the subgroup of PLWH with <200 cells/mm^3^ (n = 155) the SCD was 37% [18]. For the subset analysis of the PLWH with a left ventricular EF of 35% to 49%, SCD occurred in 39% of those with a detectable VL compared to 10% those with an undetectable VL (p < 0.001) [18]. The multivariable analysis showed that for PLWH, a history of coronary artery disease, cocaine use, and increased QRS duration were associated with an increased SCD hazard ratio, while increasing CD4 and beta-blocker use was associated with a lower SCD hazard ratio.

While Lai et al. and Yen et al. attempted to measure SCD association with HIV, the studies using the overlapping cohorts they likely has substantial misclassification bias as they includes only three ICD codes: cardiac arrest, instantaneous death cause unknown, and death within 24 hours not otherwise explained. Of these, two of the codes are non-cardiac specific, and could include deaths due to overdoses [19,20].

Tseng et al. 2021 did a 108 PLWH autopsy study of out-of-hospital SCA leading to SCD [21]. For adult PLWH, the study found a presumed SCD (which includes occult overdose) 53.3 (95% CI: 39.2–70.1) incidence rates per 100 000 person-years compared to 23.7 (95% CI: 21.7–25.9) per 100 000 person-years for adult persons without known HIV infections, resulting in an incidence rate ratio of 2.25 (95% CI: 1.37–3.70) [21]. Note that these rates do not adjust for sex and with only three female presumed SCD deaths (two occult overdoses and one intracranial hemorrhage) reported in the PLWH group and an unbalanced population of 92% male in the PLWH group and 51% male in the adult population group, female sex rate’s effect modification cannot be taken as reliable [21]. Among males, incidence rates per 100 000 person-years, were 53.0 (95% CI: 38.5–71.1) compared to 32.3 (95% CI: 29.0–35.9) for adult persons without known HIV infections, leading to an incidence rate ratio of 1.64 (95% CI: 0.91–2.95) [21]. For male confirmed sudden death from arrhythmia, PLWH had a 26.5 (95% CI: 16.6–40.1) per 100 000 person-years and 19.8 (95% CI: 17.2–22.7) for adult persons without known HIV infections with a 1.34 (95% CI: 0.62–2.87) incidence rate ratio [21]. ART status, CD4+ or VL was not adjusted for. Of the PLWH, 79% received ART and had a median CD4+ count of 475 cells/mm^3^, but the study did not account for these factors in the analysis. Tseng et al. 2021 noted that occult drug overdoses were twice as common in PLWH than in those without a known HIV infection and that one-third of apparent SCD in PLWH were due to occult drug overdoses [21]. This adds weight to the likely misclassification of SCD in the Tseng et al. 2012 study and the previously reported higher rate of SCD in PLWH. Occult drug overdoses may also be a challenge going forward for other studies with ongoing opioid crises and occult overdoses possibly more common in certain demographics.

Freiberg et al. used the 97% male PLWH USA Veterans Aging Cohort matching 1:2 using an adjusted Cox proportional hazards regression with the CD4+ or VL updated to the most recent measurement in time [22]. For veterans without HIV compared to those with HIV with CD4+ ≥ 500, the SCD adjusted risk was 1.03 (95% CI: 0.90–1.18) and with a CD4+ risk measure not reaching a significant level until CD4+ was < 200 for a risk of 1.57 (95% CI: 1.28–1.92). For VL < 500 the risk was 0.97 (95% CI: 0.87–1.09) vs VL ≥ 500 ratio of 1.70 (95% CI: 1.45–1.98).

deFilippi et al. using the data from the REPRIEVE trial of comparing pitavastain vs. a placebo to prevent cardiovascular disease in PLWH including mortality, investigated the risk of SCD [23]. It comprised of 7 769 PLWH without known cardiovascular disease and a low-to-moderate atherosclerotic cardiovascular disease risk. For all participants combined in both arms there were 25 SCDs for an incidence rate of 0.61 per 1 000 person years. Fourteen of those SCDs were in the placebo control group compared to 11 in the pitavastatin group. This study was underpowered for SCD analysis; therefore, comparison across the groups was not undertaken. SCD was determined by independent practitioners [24]. The review’s authors calculated the HR assuming a constant hazard, 0.51 SCD per 1000 person years for the pitavastatin and 0.64 SCD per 1 000 person years for the placebo group, giving a hazard rate of 1.28 (95% CI: 0.54–2.27) for the placebo group compared to the pitavastatin group.

## Discussion

With the current studies combined with the general PLWH cardiology literature, this review suggests that males living with HIV experience an increased risk of SCD when not virally suppressed. However, for male PLWH on ART with VL suppression and high CD4+ counts, the SCD risk is lower than in their unsuppressed counterparts, though still higher than the general male population. We do not yet have enough data to conduct a meta-analysis that would allow us to ascertain an accurate effect measure for the entire population. For men, a reasonable SCD risk estimation could likely be formed with the addition of two or three more well-controlled studies in sizable cohorts that utilize a homogeneity approach to SCD coding.

While SCD risk is elevated for men living with HIV, maintaining HIV-RNA plasma VL suppression and ≥200 CD4+ cells/mm^3^ counts, ideally higher, likely lowers the risk of SCD to a rate that approaches the general population risk. This study cannot quantify the risk of SCD in women living with HIV, because most of the PLWH in the eight selected studies were large majority male populations.

While it is common to focus on males in ART studies where the main HIV-infected population in many cohorts is men that have sex with men, we should still examine the effect modification of female sex, even if the cohort sizes are small, as the effect sizes of multiple studies can be combined. General population studies show a two-to-threefold higher risk of SCD in men than in women, or SCD occurring 10 years later in women than men; therefore, it is likely that PLWH SCD sex risk also has a large sex effect [25]. As the limited research in women living with HIV shares factors with the male-focused research on SCD risk, the review authors hypothesize that female PLWH SCD risk could be reduced with ART, VL suppression, and increased CD4+ counts. This hypothesis still needs to be explored. SCD risk in PLWH may also decrease for those on pitavastatin; though the evidence is yet unclear. In the phase three trial “Evaluating the Use of Pitavastatin to Reduce the Risk of Cardiovascular Disease in HIV-Infected Adults” PLWH aged 40–74 with stable antiretroviral therapy and a low-to-moderate risk of atherosclerotic cardiovascular disease were either given pitavastatin at 4 mg a day or a placebo at a 1:1 ratio for 10 865 participants [24]. With pitavastain calcium being chosen due to its lack of interactions with ART [26,27]. The incidence of major cardiovascular events or mortality, including SCD, in the pitavastatin group was 9.18 per 1 000 person-years compared to 11.63 per 1 000 person-years in the placebo group, for a hazard ratio of 0.79 (95% CI: 0.65–0.96). The reduction in the hazard ratio led to the trial being stopped early at 5.1 years. Additionally, the trial participants had minimal adverse effects. A subset analysis of the trial, as discussed in the results section, had a lower number of SCD events in the pitavastatin group compared to the placebo arm (11 vs. 14); however, due to the trial’s size or the length of follow-up, significance could not be inferred. A mechanistic substudy of the REPRIEVE found that 24 months of pitavastatin reduced arterial inflammation and lipid oxidation and reduced the volume and progression of noncalcified plaque volume [28]. Pitivastatin’s mechanism of action for PLWH is not known with certainty. It may prevent upstream causes such as coronary events (plaque rupture, myocardial infarction) and adverse cardiac remodelling potentially leading to lethal arrhythmias. As pitavastatin prescription for PLWH becomes more common, it will provide a natural experiment to determine if it lowers the risk of SCD for PLWH.

Part of the issue with quantifying the risk of SCD with VL suppression or CD4+ count is the studies reviewed typically used point in time measures for VL load or CD4+ as comparison to mortality. Prior values of VL loads or CD4+ counts impact intermediate exposures which, in turn, impact subsequent exposures, leading to possible time-dependent confounding [29]. Studies can apply methods such as marginal structural models or the g-formula to deal with time-varying exposure such as ART coverage, VL, CD4+ or censoring [29,30]. Causal inference with time-varying exposure methodology, including time-varying censoring, could be applied to provide a more accurate risk measure for PLWH.

Future studies may consider effect modification and mediation methodologies. With much of the nature of modern PLWH research being undertaken by large cohorts, causal approach methodology may prove to be more informative. Additionally, our methodology could be expanded by getting the raw data from the studies, for a more precise aggregate analysis, where the authors are willing to share the data. Future studies could look at articles where SCD occurred, but no effect measure was presented and attempt to retrieve the data from the authors to calculate a combined effect measure [31–35].

There are some limitations to consider in our review. A source of bias in this review is that most of these studies or the participants were in the United States, with three based in San Francisco. The relative homogeneity of the study populations causes concerns about the generalizability of the results. Ideally, future PLWH SCD studies will take place in sites where HIV is more commonly found in the general population, such as Africa, for a more direct comparison of PLWH SCD with the non-PLWH population, while also providing clearer evidence for female PLWH. An additional source of potential bias in this review is the studies having potential misclassification of SCD where an autopsy was not preformed such as the potential for an out of hospital neurologic death or overdose to appear to be SCD [13,14,36].

## Conclusion

The HIV field requires additional studies to verify the findings of the SCD studies to date and to delineate PLWH SCD risk factors or specific PLWH effect modifications and interactions, especially for the female sex [5]. The research presents evidence that while PLWH risk of SCD is elevated, maintaining VL suppression and ≥200 CD4+ cells/mm^3^ counts (ideally higher) lowers the risk of SCD to a rate of SCD that is approaching similarity to the general population. It seems likely that the combination of early ART initiation and no, or limited, treatment interruptions limiting viral exposure, providing pitavastatin in PLWH aged ≥40, and decreasing potential chronic inflammation, is currently the best opportunity to reduce SCD risk in PLWH; however, the data is limited and future studies will have to test this hypothesis.

## Supporting information

S1 File PRISMA 2020 checklist. (DOCX)
